# The Felicitous Success of the Subsection Molecular Oncology of International Journal of Molecular Sciences

**DOI:** 10.3390/ijms22136939

**Published:** 2021-06-28

**Authors:** Michael Welsh

**Affiliations:** Department of Medical Cell Biology, Uppsala University, Husargatan 3, P.O. Box 571, 75123 Uppsala, Sweden; Michael.welsh@mcb.uu.se

## Abstract

The evolvement of the newly started subsection IJMS molecular oncology is discussed. The breadth and depth of the journal articles is alluded to. A bright future for this subsection is anticipated, developing into a top tier cancer journal.

This editorial commemorates the publication of more than 1000 articles in the *International Journal of Molecular Sciences*, subsection *Molecular Oncology*. This is an incredible success considering that the first article was published in 2018. The scope of the subsection is posted as follows on the Journal website:

“This section of the *International Journal of Molecular Sciences* (*IJMS*) aims to rapidly publish contributions on all aspects of the most recent research on human cancers. This section lies at the interface between medicinal chemistry and oncology research. We encourage the submission of high-quality manuscripts that provide novel mechanistic insights and details of the molecular signatures of oncogenic transformation. The scope of this section includes, but is not limited to, the following:

- Biological processes like gene activity and metabolisms in the development and progression of cancers
- Mechanisms of metastasis (vascular and lymphatic dissemination and seeding)
- Molecular pathology and immunology in tumor development
- Biomarkers in diagnostic processes
- Novel therapies like drug therapy, radiotherapy, gene therapy, hormonal therapy, and immunotherapy
- Cancer vaccines”

It easily can be claimed that these anticipations have successfully been met when assessing the journal’s current track record. Thus far, 564 original articles and 420 reviews have been published, and 133 Special Issues are open or have been completed. The publications cover a broad range of various aspects of molecular oncology. Addressed are different tumor types such as prostate, lung, breast, gastric, colon, skin, leukemia and others. Molecular signaling responsible for conferring cancer phenotypes, examples of which are related to the cell cycle [1], epithelial–mesenchymal transition [2] or changes in cell–cell [3] or cell–matrix [4] interactions, mitogen-activated protein kinase (MAPK) [5], phosphatidyl inositol 3′ kinase (PI3K) [6], mammalian target of rapamycin (mTOR) [7], Wnt [8], p53 [9], Myc [10], cytokines/chemokines [11,12], gene mutations [13], epigenetics [14] and miRNA [15], are addressed in a broad and/or deep manner.

Recent topics of cancer research are well represented. Novel diagnostics and biomarkers [16,17], the use of exosomes as biomarkers and/or relevance for the development of disease [18,19], and the influence of the microbiome for cancer initiation and progression [20] are addressed in abundance. Immuno-oncology [12] and tumor angiogenesis [21] have had numerous contributions, and epidemiological papers or other translational approaches are plentiful [22,23]. Papers addressing novel therapies including cancer vaccines are also frequently represented [5,9,10,24].

In summary, the journal aims have all been successfully fulfilled during the short time it has been active and the future seems bright. The challenge lying ahead is to spread the excellence of *IJMS Molecular Oncology* in the cancer community, making it widely accepted as a top tier journal for the publication of the most recent cancer research of the highest quality.
